# Complete chloroplast genome sequence and phylogenetic analysis of *Mallotus paniculatus* (Lam.) Müll. Arg. (*Euphorbiaceae*)

**DOI:** 10.1080/23802359.2022.2107442

**Published:** 2022-08-08

**Authors:** Zhuowei Li, Nong Zhou, Ming Liu, Fuqiang Yin

**Affiliations:** a College of Environmental and Chemical Engineering, Chongqing Three Gorges University, Chongqing, China; b College of Biological and Food Engineering, Chongqing Three Gorges University, Chongqing, China

**Keywords:** Complete chloroplast genome, *Mallotus paniculatus* (Lam.) Müll. Arg., phylogenetic analysis, sequence matching

## Abstract

*Mallotus paniculatus* (Lam.) Müll. Arg. 1865 (*Euphorbiaceae*) is a shrub or small tree with medicinal properties that is distributed across Southeast Asia. In this study, we sequenced the complete chloroplast genome of *M. paniculatus* to study phylogenetic relationships within the family *Euphorbiaceae* Juss. The complete chloroplast genome of *M. paniculatus* was 164,455 bp in length, with an overall GC content of 35.3%. It was found to consist of a long single copy region of 89,021 bp, a small single copy region of 18,524 bp, and a pair of inverted repeats of 28,455 bp. Results indicated that the chloroplast genome contains a total of 131 genes, including 78 protein-coding genes, 37 tRNA genes, eight rRNA genes, and eight pseudogenes. The phylogenetic tree showed that *M. paniculatus* is closely related to *Mallotus japonicus* and *Mallotus peltatus*.

*Mallotus paniculatus* (Lam.) Müll. Arg. is a shrub or small tree belonging to the spurge family (*Euphorbiaceae* Juss.), which was first described by Lamarck in 1865 (Lamarck 1865). It is primarily distributed in the Chinese provinces of Yunnan, Guizhou, Guangxi, Guangdong, Hainan, Fujian, and Taiwan, where it grows in thickets at altitudes of 50–1300 m above sea level. In traditional medicine, the leaves and roots of *M. paniculatus* are often used as a drug and have a slightly bitter, astringent, and flat taste (Qiu 1996). The leaves are used for heat-clearance, damp-elimination, and detoxification, as well as to relieve pain and stop bleeding, whereas the roots have anti-inflammatory and astringent properties (Rivière et al. 2010; Wang et al. 2013; Zhu and Ma 2014). In Guangxi province, the plant is used to treat dysentery, otitis media, and other ailments. Previous studies on this species have focused on its chemical composition, morphological characteristics, and biological activities. However, no studies on the molecular biological properties of *M. paniculatus* have been published. Here, we studied the sequence of the chloroplast genome of *M. paniculatus* and revealed the phylogenetic relationship between this plant and other species of the *Euphorbiaceae* family.

Fresh leaves of *M. paniculatus* were collected in the city of Fangchenggang, Guangxi, China. (107.98° E, 22.15° N). The specimens were identified by Nong Zhou (erhaizn@126.com) and deposited into the herbarium of the Chongqing Three Gorges University (https://www.sanxiau.edu.cn) under the voucher number ZN20210315. Total genomic DNA was extracted using the improved CTAB method (Doyle 1987; Yang et al. 2014) and sequenced with the Illumina HiSeq 2500 platform (Novogene, Tianjin, China). High-throughput sequencing generated approximately 5.3 Gb of raw data. The default settings of the Trimmomatic software (v. 0.32) were used to screen the original data (Bolger et al. 2014). Then, the obtained clean reads were assembled into circular contigs using the GetOrganelle toolkit (Jin et al. 2020) with *Mallotus peltatus* (NC_047284) as the reference. Finally, the cpDNA was annotated using the Dual Organellar Genome Annotator GeSeq (Tillich et al. 2017) and CpGAVAS2 (Shi et al. 2019). The chloroplast genome was submitted to GenBank (accession number: MZ597547).

The total length of the chloroplast genome was 164,455 bp, and the total GC content was 35.3%. The chloroplast genome of *M. paniculatus* showed a typical quadripartite structure, including a pair of inverted repeats of 28,455 bp each, separated by a small single copy region of 18,524 bp and a long single copy region of 89,021 bp. The sequence of the chloroplast genome revealed a total of 131 genes, including 78 protein-coding genes, 37 tRNA genes, and eight rRNA genes. Further, we identified a total of eight pseudogenes (*rps16*, *rbcL*, *petB*, *ndhF*, *ndhD*, *ndhG*, and a pair of reverse repeats of *ycf1*).

To study their phylogenetic relationships, the chloroplast genome sequences of 26 *Euphorbiaceae* species and two *Daphniphyllaceae* species were downloaded from the GenBank database. Sequence alignment was performed using MAFFT (v. 7.427) (Katoh and Standley 2013). A maximum-likelihood phylogenetic tree was constructed using RAxML (Stamatakis 2014), with 1000 bootstrap replicates, and by applying the GTRGAMMAI model. The maximum likelihood phylogenetic tree showed that among the species analyzed, *M. japonicus* and *M. peltatus* were the closest relatives of *M. paniculatus* (Figure 1). This study provides a scientific basis to exploit these resources and a foundation for further phylogenetic analyses of *M. paniculatus*.

**Figure 1.**
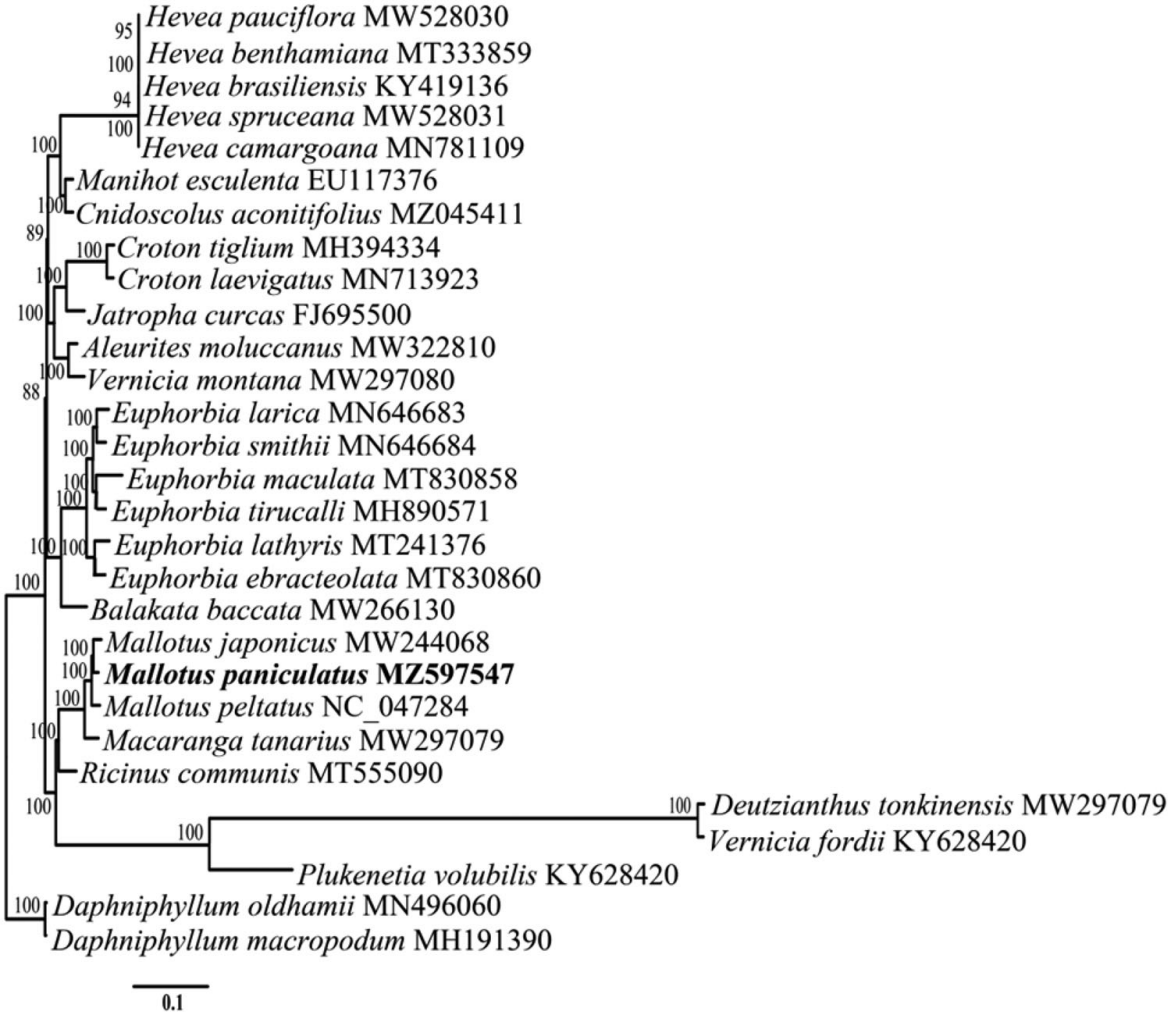
Maximum-likelihood phylogenetic tree based on the complete chloroplast genome sequences of 29 different plant species; *Daphniphyllum oldhamii* and *Daphniphyllum macropodum* were used as the outgroups. Bootstrap values (1000 replicates) are shown next to the nodes.

## Data Availability

The data that support the findings of this study are openly available in GenBank of NCBI at https://www.ncbi.nlm.nih.gov, accession number MZ597547. The associated BioProject, SRA, and BioSample numbers are PRJNA758621, SRR15663411, and SAMN21031881, respectively.

## References

[CIT0001] Bolger AM, Lohse M, Usadel B. 2014. Trimmomatic: a flexible trimmer for Illumina sequence data. Bioinformatics. 30(15):2114–2120. 24695404 10.1093/bioinformatics/btu170 PMC4103590

[CIT0002] Doyle J. 1987. A rapid DNA isolation procedure for small quantities of fresh leaf tissue. Phytochem Bull. 19:11–15.

[CIT0003] Jin J-J, Yu W-B, Yang J-B, Song Y, dePamphilis CW, Yi T-S, Li D-Z. 2020. GetOrganelle: a fast and versatiletoolkit for accurate de novo assembly of organelle genomes. Genome Biol. 21(1):241. 32912315 10.1186/s13059-020-02154-5 PMC7488116

[CIT0004] Katoh K, Standley DM. 2013. MAFFT multiple sequence alignment software version 7: improvements in performance and usability. Mol Biol Evol. 30(4):772–780. 23329690 10.1093/molbev/mst010 PMC3603318

[CIT0005] Lamarck JB. 1865. *Mallotus paniculatus* (Lam.) Müll.Arg. Linnaea. 34(2):189.

[CIT0006] Qiu HX. 1996. Flora Reipublicae Popularis Sinicae (44-2). Beijing: Science Press; p. 35–36.

[CIT0007] Rivière C, Nguyen Thi Hong V, Tran Hong Q, Chataigné G, Nguyen Hoai N, Dejaegher B, Tistaert C, Nguyen Thi Kim T, Vander Heyden Y, Chau Van M, et al. 2010. Mallotus species from Vietnamese mountainous areas: phytochemistry and pharmacological activities. Phytochem Rev. 9(2):217–253.

[CIT0008] Shi L, Chen H, Jiang M, Wang L, Wu X, Huang L, Liu C. 2019. CPGAVAS2, an integrated plastome sequence annotator and analyzer. Nucleic Acids Res. 47(W1):W65–W73. 31066451 10.1093/nar/gkz345 PMC6602467

[CIT0009] Stamatakis A. 2014. RAxML version 8: a tool for phylogenetic analysis and post-analysis of large phylogenies. Bioinformatics. 30(9):1312–1313. 24451623 10.1093/bioinformatics/btu033 PMC3998144

[CIT0010] Tillich M, Lehwark P, Pellizzer T, Ulbricht-Jones ES, Fischer A, Bock R, Greiner S. 2017. GeSeq-versatile and accurate annotation of organelle genomes. Nucleic Acids Res. 45(W1):W6–W11. 28486635 10.1093/nar/gkx391 PMC5570176

[CIT0011] Wang WJ, Jiang JH, Chen YG. 2013. Steroids from *Mallotus paniculatus*. Chem Nat Compd. 49(3):577–578.

[CIT0012] Yang JB, Li DZ, Li HT. 2014. Highly effective sequencing whole chloroplast genomes of angiosperms by nine novel universal primer pairs. Mol Ecol Resour. 14(5):1024–1031. 24620934 10.1111/1755-0998.12251

[CIT0013] Zhu CL, Ma JX. 2014. Chemical constituents of *Mallotus paniculatus*. Zhong Yao Cai. 37(8):1385–1387. 25726646

